# Multisite Autism Spectrum Disorder Classification Using Convolutional Neural Network Classifier and Individual Morphological Brain Networks

**DOI:** 10.3389/fnins.2020.629630

**Published:** 2021-01-28

**Authors:** Jingjing Gao, Mingren Chen, Yuanyuan Li, Yachun Gao, Yanling Li, Shimin Cai, Jiaojian Wang

**Affiliations:** ^1^School of Information and Communication Engineering, University of Electronic Science and Technology of China, Chengdu, China; ^2^School of Computer Science and Engineering, University of Electronic Science and Technology of China, Chengdu, China; ^3^Key Laboratory for NeuroInformation of the Ministry of Education, School of Life Sciences and Technology, University of Electronic Science and Technology of China, Chengdu, China; ^4^School of Physics, University of Electronic Science and Technology of China, Chengdu, China; ^5^School of Electrical Engineering and Electronic Information, Xihua University, Chengdu, China; ^6^Center for Language and Brain, Shenzhen Institute of Neuroscience, Shenzhen, China

**Keywords:** autism spectrum disorder, individual morphological covariance brain network, convolutional neural network, gradient-weighted class activation mapping, structural MRI

## Abstract

Autism spectrum disorder (ASD) is a range of neurodevelopmental disorders with behavioral and cognitive impairment and brings huge burdens to the patients’ families and the society. To accurately identify patients with ASD from typical controls is important for early detection and early intervention. However, almost all the current existing classification methods for ASD based on structural MRI (sMRI) mainly utilize the independent local morphological features and do not consider the covariance patterns of these features between regions. In this study, by combining the convolutional neural network (CNN) and individual structural covariance network, we proposed a new framework to classify ASD patients with sMRI data from the ABIDE consortium. Moreover, gradient-weighted class activation mapping (Grad-CAM) was applied to characterize the weight of features contributing to the classification. The experimental results showed that our proposed method outperforms the currently used methods for classifying ASD patients with the ABIDE data and achieves a high classification accuracy of 71.8% across different sites. Furthermore, the discriminative features were found to be mainly located in the prefrontal cortex and cerebellum, which may be the early biomarkers for the diagnosis of ASD. Our study demonstrated that CNN is an effective tool to build the framework for the diagnosis of ASD with individual structural covariance brain network.

## Introduction

Autism spectrum disorder (ASD) encompasses a range of neurodevelopmental disorders. The core symptoms of ASD comprise abnormal emotional regulation and social interactions, restricted interest, repetitive behaviors, and hypo-/hyperreactivity to sensory stimuli (Guze, 1995). Many individuals with autism spectrum disorder usually exhibit impairments in learning, development, control, and interaction, as well as some daily life skills. ASD causes heavy economic burden for the patients’ families and the society. It is urgent to establish an early and accurate diagnosis framework to identify ASD patients from typical controls (TC). Recently, noninvasive and *in vivo* neuroimaging techniques have become an area of intense investigation to explore the auxiliary diagnostic for ASD.

A variety of neuroimaging modalities, such as structural MRI (sMRI), diffusion MRI, functional MRI, magnetoencephalography, electroencephalography, and electrocorticography, are widely adopted to uncover patterns in both brain anatomical structure and function. Structural MRI provides abundant measures to delineate the structural properties of the brain (Wang et al., 2017, 2018, 2019; Wu et al., 2017; Xu et al., 2019). Although there were many controversial findings regarding brain structural changes in individuals with autism (Chen et al., 2011), to identify ASD based on sMRI still received a great deal of attention from researchers. Wang et al. (2016) linearly projected gray matter (GM) and white matter (WM) features extracted from sMRI onto a canonical space to maximize their correlations. With the projected GM and WM as features, they achieved the classification accuracy of 75.4% with the data scanned at New York University (NYU) Langone Medical Center from ABIDE. Recently, the classification accuracy for ASD is up to 90.39%, which is obtained with sMRI constrained to the same single-site dataset (Kong et al., 2019). Although previous studies achieved high classification accuracy, almost all these results were obtained from a single site, and reproducibility and generation in a multisite remain uncertain (Button et al., 2013).

It is well accepted that multisite datasets can represent greater variance of disease and control samples to establish more stable generalization models for replication across different sites, participants, imaging parameters, and analysis methods (Nielsen et al., 2013). Thus, there are some studies focused on larger sample sizes from a multisite. However, the classification accuracy drops significantly due to the complexity and heterogeneity of ASD (Nielsen et al., 2013). In a study by Zheng et al. (2018), the elastic network was utilized to quantify corticocortical similarity based on seven morphological features on 132 selected subjects from four independent sites of the ABIDE dataset. Although the classification performance of this study was 78.63%, the samples used in this study only cover a small portion of the ABIDE dataset. Additionally, there are few other studies that use larger datasets from ABIDE but with low accuracy. For instance, Haar et al. (2016) used linear and quadratic (nonlinear) discriminant analyses to classify ASD and control subjects based on structural measures of the quality-controlled samples from ABIDE, but the accuracies were only 56 and 60% when using subcortical volumes or cortical thickness measures. Katuwal et al. (2015) used random forest (RF), support vector machine (SVM), and gradient boosting machine (GBM) to classify 373 ASD from 361 TC male subjects from the ABIDE database and obtained 60% classification accuracy. The accuracy was further improved to 67% when IQ and age information were added to morphometric features. Although the existing approaches can obtain high classification accuracy based on sMRI measures in a single site or with a small number of subjects, an acceptable method to achieve high classification accuracy across different sites with different scanning paradigms is still needed.

The low classification accuracy for different sites may mainly result from the following reasons. First, the data collected from different sites expand the variances of structural measures, which increases the difficulty in learning high accuracy classifiers with such data. Second, the brain is an integrative and dynamic system for information processing between brain regions (Vértes and Bullmore, 2014), yet most of the existing methods only extract independent local morphological features of different brain areas with sMRI and did not consider the interregional morphological covariant relationships. Third, although some deep neural network classifiers were used in ASD/TC classification by transforming the features to a one-dimensional vector followed by features selection algorithm, the classification results are hard to interpret in the absence of the contributions of the classification features leading to lack of clinical significance. In view of the abovementioned problems, to further explore an efficient classification method is essential to establish the ASD diagnosis model. Here, we combine a deep learning classifier and gradient-weighted class activation mapping (Grad-CAM) (Selvaraju et al., 2020) based on morphological covariance brain networks to identify ASD patients from TC with all the ABIDE dataset. We first constructed the individual-level morphological covariance brain networks, and the interregional morphological covariance values were used as the input feature for the classifier. Next, the convolutional neural network (CNN) classifier with Grad-CAM is applied to differentiate ASD from TC and to identify features contributing the largest for the classification in our framework.

## Materials and Methods

### The ABIDE Dataset

Data used in this study are accessed from a large open access data repository, Autism Brain Imaging Data Exchange I (ABIDE^1^), which came from 17 international sites with no prior coordination (Di Martino et al., 2014). ABIDE includes structural MRI, corresponding rs-fMRI, and phenotype information for individuals with ASD and TC and allows for replication, secondary analyses, and discovery efforts. Although all data in ABIDE were collected with 3 T scanners, the sequence parameters as well as the type of scanner varied across sites. In this paper, we used the structural MR images of 518 ASD patients and 567 age-matched normal controls (ages 7–64 years, median 14.7 years across groups) aggregated from all 17 international sites. The key phenotypical information is summarized in Table 1. As seen from Table 1, the variation in age range across samples varied greatly, and most of the ASD subjects are male with 25% of the sites excluding females by design.

**TABLE 1:** Demographic information of subjects with autism spectrum disorder (ASD) and typical controls (TC).

Site	Autism spectrum disorder (ASD)			Typical controls (TC)	
	Number of subjects	Age (years)	ADOS	Number of subjects	Age (years)
CALTECH	19 (15 M/4 F)	27.4	13.1	18 (14 M/4 F)	28
CMU	14 (11 M/3 F)	26.4	13.1	13 (10 M/3 F)	26.8
KKI	20 (16 M/4 F)	10.0	12.5	28 (20 M/8 F)	10
LEUVEN	29 (26 M/3 F)	17.8	*	34 (29 M/5 F)	18.2
MAX MUN	24 (21 M/3 F)	26.1	9.5	28 (27 M/1 F)	24.6
NYU	75 (65 M/10 F)	14.7	11.4	100 (74 M/26 F)	15.7
OHSU	12 (12 M/0 F)	11.4	9.2	14 (14 M/0 F)	10.1
OLIN	19 (16 M/3 F)	16.5	14.1	15 (13 M/2 F)	16.7
PITT	29 (25 M/4 F)	19.0	12.4	27 (23 M/4 F)	18.9
SBL	15 (15 M/0 F)	35.0	9.2	15 (15 M/0 F)	33.7
SDSU	14 (13 M/1 F)	14.7	11.2	22 (16 M/6 F)	14.2
STANFORD	19 (15 M/4 F)	10.0	11.7	20 (16 M/4 F)	10
TRINITY	22 (22 M/0 F)	16.8	10.8	25 (25 M/0 F)	17.1
UCLA	54 (48 M/6 F)	13.0	10.9	44 (38 M/6 F)	13.0
UM	66 (57 M/9 F)	13.2	*	74 (56 M/18 F)	14.8
USM	46 (46 M/0 F)	23.5	13.0	25 (25 M/0 F)	21.3
YALE	28 (20 M/8 F)	12.7	11.0	28 (20 M/8 F)	12.7

### Data Preprocessing

All structural MR images used in our work were preprocessed by the deformable medical image registration toolbox—DRAMMS (Ou et al., 2011). DRAMMS is a software package designed for 2D-to-2D and 3D-to-3D deformable medical image registration tasks. The preprocessing procedures in our study include cross-subject registration, motion correction, intensity normalization, and skull stripping. Especially, T1W MRI images in different sites were registered to the SRI24 atlas (Rohlfing et al., 2010) for the morphological covariance brain network mapping.

### Individual-Level Morphological Covariance Brain Networks

The morphological features of the human brain have long been characterized by structural MRI. In our study, the individual-level morphological covariance brain network (Wang et al., 2016) is used to estimate interregional structural connectivity to characterize the interregional morphological relationship. The detailed construction procedures were described below. First, after preprocessing, the structural T1 images were segmented into cerebrospinal fluid (CSF), WM, and GM by the multiplicative intrinsic component optimization (MICO) method (Li et al., 2014). Next, a GM volume map was obtained for each participant in template space. Second, the large-scale morphological covariance brain network for each participant was constructed based on their GM volume images according to a previous study (Wang et al., 2016). A brain network is usually comprised of a collection of nodes and edges, wherein the network nodes are defined as different regions in SRI24 atlases and the edge is defined as the interregional similarity in the distribution of the regional GM volume. The SRI24 atlas (Rohlfing et al., 2010) parcellates the whole brain into 116 subregions and 58 subregions in each hemisphere. Because of low signal-to-noise ratio and blank values of gray matter volume in Vermis during network analysis, we excluded eight areas in the Vermis (the cerebellar Vermis labeled from 109 to 116) to ensure the reliability of our study. Finally, a 108 × 108 matrix was obtained for each subject for further analyses. To be specific, the edge of the individual network is calculated as follows: the kernel density estimation (KDE) (Rosenblatt, 1956) is firstly used to estimate the probability density function (PDF) of the extracted GM volume values as Eq. (1).

(1) $$P\left(i\right)=\frac{f_{h}\left(i\right)}{\sum_{j=1}^{N}f_{h}\left(j\right)}$$

where *P*(*i*) is the PDF of the *i*-th brain area, *N* is the total number of regions, and *f*_*h*_(*i*) is the kernel density of the *i*-th area defined as (Wang et al., 2016):

(2) $$f_{h}\left(i\right)=\frac{1}{nh}\sum_{j=1}^{N}K\left(\frac{v\left(i\right)-v\left(j\right)}{h}\right)$$

where *K*(.) is a non-negative function that integrates to one and has mean zero, *h >* 0 is a smoothing parameter called the bandwidth, and *v*(*i*) is the GM volume value of the *i*-th ROI.

The variation of the KL divergence (KLD) is calculated subsequently from the above PDFs as Eq. (3):

(3) $$D_{\text{KL}}\left(P,Q\right)=\sum_{i=1}^{N}\left(P\left(i\right)\log\frac{P\left(i\right)}{Q\left(i\right)}+Q\left(i\right)\log\frac{Q\left(i\right)}{P\left(i\right)}\right)$$

where *P* and *Q* are the PDFs of different ROI. The network edge is formally defined as the structural connectivity between two regions and is quantified by a KL divergence-based similarity (KLS) measure (Kong et al., 2014) with the calculated variation of KLD. Thus, the similarity matrix can be defined as:

(4) $$\text{KLS}\left(P,Q\right)=e^{-D_{\text{KL}}\left(P,Q\right)}$$

For more details of the calculation process, refer to the previous literature (Wang et al., 2016).

### Convolutional Neural Network Classifier

**FIGURE 1:**
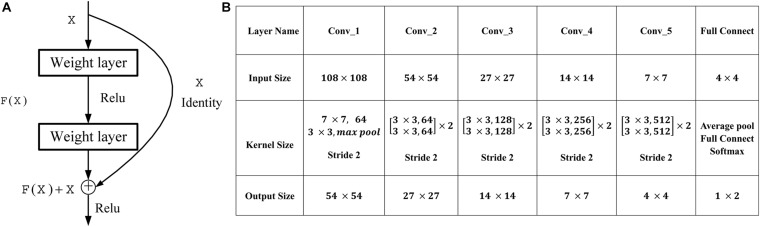
Residual learning and architectures for our work. **(A)** The formulation of *F*(*X*) + *X* can be realized by feedforward neural networks with shortcut connections. **(B)** Architectures for our work. The downsampling is performed by Conv_1, Conv_2, Conv_3, Conv_4, and Conv_5 with a stride of 2.

Deep CNNs have led to a series of breakthroughs for image classification (Liu et al., 2017, 2018). The deep residual networks (ResNet) (He et al., 2016) have recently achieved state-of-the-art on challenging computer vision tasks, which consists of an ensemble of basic residual unit. According to the study of He et al. (2016), ResNet tries to learn both local and global features *via* skip connections combining different levels to overcome the incapability of integrating different level features found in plain networks, as seen in Figure 1A. Here, *X* is the input of the residual unit, and *F*(*X*) denotes the residue mapping of the stacked convolution layers. The formulation of *F*(*X*) + *X* can be realized by feedforward neural networks with shortcut connections. Thus, a simple identity mapping directly connects the input and output layers by using ResNet with the skip connection. In our work, five bottlenecks are used to perform the classification, and the detailed architecture of ResNet used in our work is shown in Figure 1B. The binary cross-entropy (BCE) cost function is used as the loss function of our work in the residual learning, which is defined as follows:

(5) $$L=-\left(y*\log\left(\overset{\wedge }{y}\right)+\left(1-y\right)*\log\left(1-\overset{\wedge }{y}\right)\right)$$

where *y* is the ground-truth label, and $\hat{y}$ is the prediction result of our work.

### Grad-CAM

The Grad-CAM is able to produce “visual explanations” for decisions from a large class of CNN-based models and makes them more transparent (Selvaraju et al., 2017, 2020). It uses the gradient information flowing into the last convolutional layer of CNN to assign importance values to each neuron for a particular decision of interest to avoid the model structure modification and refrainment to keep both interpretability and accuracy (Selvaraju et al., 2020). Also, the produced localization map highlights the important regions in the image for predicting the concept. It is an important tool for users to evaluate and place trust in classification systems (Selvaraju et al., 2020). In this work, we only focus on the explaining output layer decisions by identifying the contributions of the classification features to help researchers focus on the highlighted regions and trust the model. The gradient of the score *y*^*c*^ for class *c* is computed with respect to feature map activations *A*^*k*^ of a convolutional layer. In the study by Selvaraju et al. (2020), the neuron importance weights $\alpha_{k}^{c}$ are defined as:

(6) $$\alpha_{k}^{c}=\frac{1}{Z}\sum_{i}\sum_{j}\frac{\partial y^{c}}{\partial A_{i,j}^{k}}$$

where $A_{i,j}^{k}$ is the feature map activations *A*^*k*^ indexed by *i* and *j*.

Then, a ReLU function is applied to a weighted combination of forward activation maps to obtain the Grad-CAM (Selvaraju et al., 2020):

(7) $$L_{\mathrm{Grad}-\mathrm{CAM}}^{c}=\text{ReLU}\left(\sum_{k}\alpha_{k}^{c}A^{k}\right)$$

Finally, the heat map highlighting the regions with a positive influence on ASD/TC classification is obtained by upsampling $L_{\text{Grad-CAM}}^{c}$.

### Implementation

**FIGURE 2:**
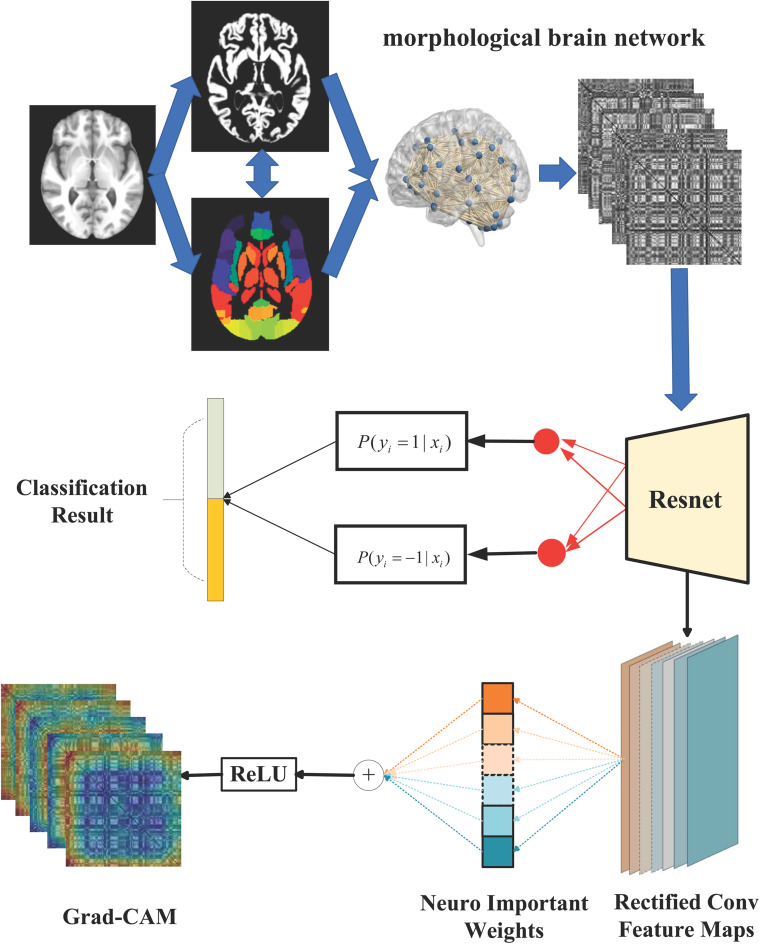
The overall flow chart of our study. Briefly, the individual-level morphological covariance brain network is first constructed according to the SRI24 atlas and gray matter volume map of each subject, as shown in the dashed boxes. The above morphological covariance brain network is used as the input feature for the classifier. Subsequently, the ResNet network is used to perform the ASD/TC classification, as shown in the middle of this figure. Meanwhile, the Grad-CAM is combined with the ResNet-based architecture based on the rectified convolution feature maps from the ResNet to obtain the heat map for explaining model decisions, as shown at the bottom of this figure.

An overview of the proposed ASD/TC classification framework in our work is shown in Figure 2. We first constructed the individual-level morphological covariance brain network according to the SRI24 atlas as the input images for the classification. Subsequently, the ResNet network is used to perform the classification. Meanwhile, the importance value to each neuron is obtained from Grad-CAM to explain model decisions. Specially, our implementation for ASD/TC classification based on the morphological covariance brain networks performs the following practice. The size of input image for ResNet is 108 × 108, and the crop is not needed to be sampled from an image or its horizontal flip. We adopt batch normalization (Ioffe and Szegedy, 2015) right after each convolution and before activation based on a previous study (Ioffe and Szegedy, 2015). Moreover, we do not use dropout (Hinton et al., 2012) which is the practice of a previous study (Ioffe and Szegedy, 2015). We initialized the weights randomly and trained all plain/residual nets from scratch. We use the Adam optimization method (Zhang et al., 2016) with a minibatch size of 32 to minimize the BCE in this study. Also, the learning rate is settled as 1*e*-5 in our ResNet work. Furthermore, we use a 10-fold cross-validation strategy in specific implementation processes and repeat 20 times to evaluate our proposed method. Specifically, all subjects used in our work are randomly equally partitioned into 10 groups defined as {*S*_1_, *S*_2_, *S*_3_,…, *S*_10_} in each classification process. Group *S*_10_ is usually set as the testing set and the other nine groups are further randomly equally repartitioned into 10 subgroups, one of which is selected as the validation set and the other nine subgroups used as the training data.

## Results

**FIGURE 3:**
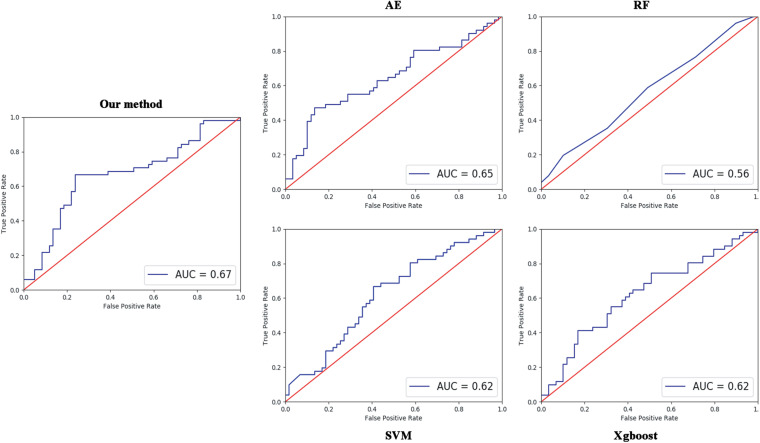
Comparisons between our method and other methods for classification. The area under the curve (AUC) values were used to assess the classification performances for our method, AE, RF, SVM, and XgBoost.

**FIGURE 4:**
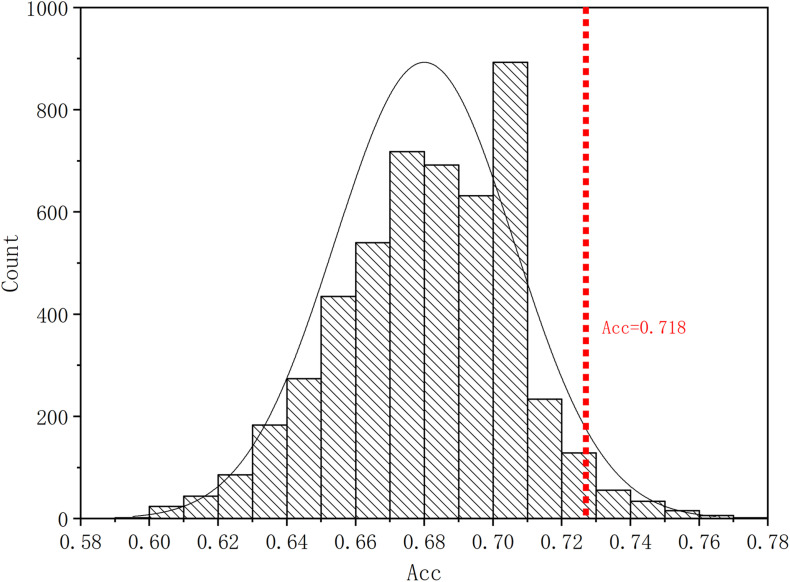
The histogram of accuracy of the permutation test. The permutation test with 5,000 times was used to evaluate the significance of our method. The accuracy of our method (0.718) is indicated by the red dotted line. The classification accuracy is higher than 95% of the permutated accuracy values.

Four parameters, namely accuracy (ACC), sensitivity (SEN), specificity (SPE), and F1 score, are calculated to evaluate the performance of our proposed ASD/TC classification framework. The deep convolutional neural network used in our work achieved a mean classification accuracy of 71.8%, mean sensitivity value of 81.25%, specificity value of 68.75%, and F1 score of 0.687 from cross-validation. Our results improved the mean classification accuracy of the state-of-the-art from 70 to 71.8% in the ABIDE data, and the former accuracy is obtained by DNN based on fMRI in ABIDE (Heinsfeld et al., 2018). To evaluate our results obtained with the deep convolutional neural network, the performance of our model is compared with the results of classifiers trained using RF (Vapnik, 1998), SVM (Ho, 1995), XgBoost (XGB) (Chen and Guestrin, 2016), and autoencoder (AE). With the purpose of using 2D input data for subject classification by these conventional machine learning methods, a vector of features is firstly retrieved by flattening the 2D morphological covariance brain network (i.e., collapse it in a one dimension vector). The number of resultant features is 11,664, which is computed by 108 × 108. Evaluation of all the models is based on a 10-fold cross-validation schema, which mixes data from all 17 sites while keeping the proportions between different sites. The results of comparing these methods are reported in Table 2. Furthermore, the performance of these classifiers was assessed by the area under the curve (AUC) values shown in Figure 3. Our proposed framework has the best performances in classifying ASD from TC with the highest ACC, SEN, F1 score, and AUC values compared with the other methods. Furthermore, the permutation test with 5,000 times is used to evaluate the significance of the prediction accuracy. During the permutation testing, 20% of the labels of the samples are changed randomly in each time. The histogram of accuracy of the permutation test is shown in Figure 4. The accuracy of our method (0.718) is indicated by the red dotted line. As shown in Figure 4, the 71.8% accuracy of our method is higher than 95% of the permutated accuracy values.

**TABLE 2:** Comparison of the classification performances between our method and other methods.

Method	Accuracy	Sensitivity	Specificity	F1 score
Our method	0.718182	0.8125	0.6875	0.686869
AE	0.672727	0.6875	0.875	0.571429
RF	0.536364	0.352941	0.694915	0.413793
SVM	0.618182	0.529412	0.694915	0.5625
XgBoost	0.609091	0.529412	0.677966	0.556701

**FIGURE 5:**
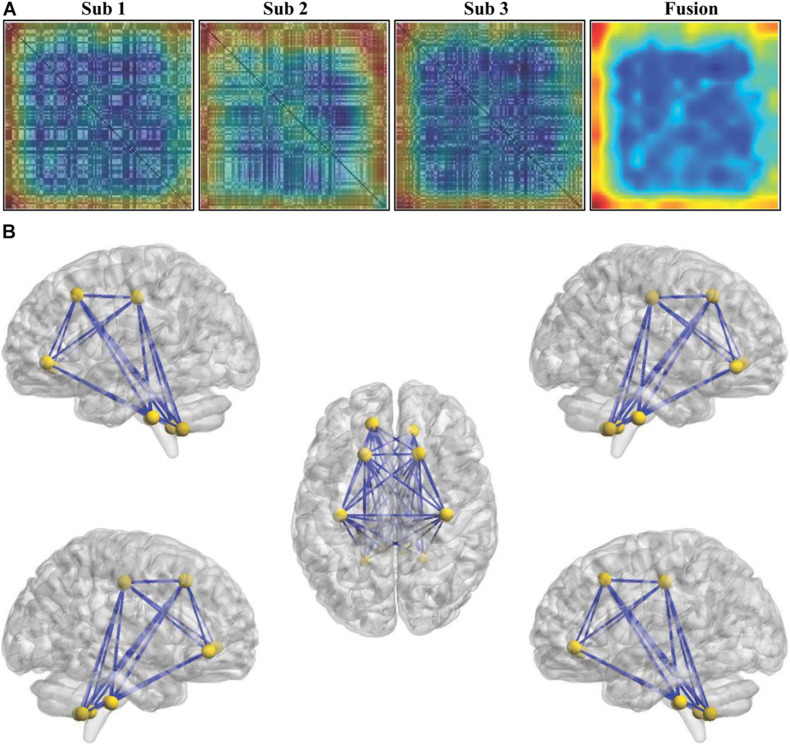
Weights of the features for classification. **(A)** The individual and fused connectivities supporting the correct classification of ASD patients were mapped using the Grad-CAM visualizations for our ResNet framework. The red regions correspond to high score for ASD class. **(B)** The top 12 regions and the corresponding connectivities which have the largest contribution correctly classifying ASD patients were identified.

To determine the weight values of the features contributing to classification, the Grad-CAM method was adopted, and the weight of each connectivity was obtained. The individual and fused connectivities supporting the correct classification of ASD patients using Grad-CAM visualizations for our ResNet framework are shown in Figure 5A. In order to make the fusion of Grad-CAM more transparent and explainable, we selected the covariance connectivities with the largest contribution to classification. The largest contributions of the connectivities were determined by identifying the weights above the mean + 3SD. Finally, 63 connectivities between 12 different regions were found using the fused Grad-CAM approach. The top 12 regions correspond to the bilateral precentral gyrus (left and right), superior frontal gyrus, orbital part of the superior frontal gyrus, and cerebellum 8–10 (see Figure 5B).

## Discussion

As far as we know, the great majority of machine learning or deep learning methods used to identify ASD patients from controls are mainly based on the resting-state fMRI. Although fMRI constructs individual brain networks by estimating interregional functional connectivity, those networks are made by the graph theory analytical method, which are only efficient for imaging data with 4D time series. However, given the great individual variability of fMRI, sMRI and its derived measures with high reproducibility have been widely used for disease classification. Although some previous studies extracted local conventional morphological features, such as gray matter volume, thickness, or volume of different regions from sMRI for machine learning, the relationship between structural properties of different regions has not been explored since the coordinated patterns of the local morphological features between regions are important for cognitive development (Bullmore and Bassett, 2010; Vértes and Bullmore, 2014). Thus, the construction of a structural covariance network with sMRI to explore individual brain topological organization and to investigate its alterations or abnormalities under both healthy and pathological conditions has attracted increasing attention. The whole-brain morphological network at the individual level based on sMRI characterizes the topological organizations at both the global and nodal levels (Wang et al., 2016). Thus, the individual-level morphological brain networks can better reflect individual behavior differences in both typical and atypical populations than the group-level morphological network. Moreover, compared with the local regional measures with sMRI, the individual morphological covariance network can provide more features to meet the requirements for the number of features during deep learning training. Thus, combining the morphological covariance network and deep learning will open a new avenue for future studies with sMRI.

In recent works, deep learning algorithms improve the classification accuracy in the identification of ASD versus TC. However, they are usually treated as “black-box” methods because of the lack of understanding of their internal functions (Lipton, 2016). The black-box methods cannot explain their predictions in a way that humans can understand. To fix this problem, the Grad-CAM technique uses gradients of the target concept (identification of ASD in a classification network for our work) and produces a coarse localization map to identify the weight of each feature of the image during classification or prediction, which produces “visual explanations” for decisions from the CNN-based models and makes them to be more transparent and explainable (Selvaraju et al., 2020). Compared with other visualization techniques, Grad-CAM can highlight the important connectivities in the morphological covariance brain networks for discriminating ASD without model architectural changes or retraining. Thus, Grad-CAM combined with our classification model provides a reference for future study to determine the important features in deep learning framework.

Using the Grad-CAM method, we found that the morphological covariance between frontal and cerebellar areas has the largest contribution for classification. The frontal areas include the precentral gyrus, superior frontal gyrus, and orbital part of the superior frontal gyrus. For the cerebellum, cerebellum 8, 9, and 10 were found to contribute greatly for classification. The precentral gyrus and cerebellum have been widely demonstrated to be associated with motor processing and integration of sensory information. Thus, the structural covariance between the precentral gyrus and cerebellum suggested that they may be related to the rigid, stereotyped, and repetitive behaviors in ASD (Mei et al., 2020). Furthermore, we also found the structural covariance connectivities among the superior frontal gyrus, orbital part of the superior frontal gyrus, and cerebellum contributing largely to the classification. The cerebellum participates not only in motor functions but also in emotion, memory, language, and social cognition processing (Strick et al., 2009; Buckner, 2013). The superior frontal gyrus has been demonstrated to be involved in social cognition (Monk et al., 2009; Li et al., 2013). Thus, the superior frontal gyrus and its orbital part and the cerebellum may be related to social cognition processing in ASD. Given that rigid, stereotyped, and repetitive behaviors and impaired social cognition are the core symptoms of ASD, our findings further demonstrated the reliability and feasibility of our proposed method for ASD classification. Also, our proposed method may advance establishing the framework for early diagnosis of ASD.

## Conclusion

In this study, we proposed a convolutional neural network framework based on the individual-level morphological covariance brain network for ASD diagnosis. We found that our proposed method outperformed other classification methods for the classification of ASD in a multisite. Moreover, using Grad-CAM, we can identify the weight of each feature for classification, which solves the black-box problems of deep learning. Our study proposes a new paradigm for ASD classification that has a good performance in multisite datasets and will facilitate establishing the diagnosis framework for ASD.

## Data Availability Statement

The raw data supporting the conclusions of this article will be made available by the authors, without undue reservation.

## Ethics Statement

The studies involving human participants were reviewed and approved by the St. James’s Hospital/AMNCH (ref: 2010/09/07) and the Linn Dara CAMHS Ethics Committees (ref: 2010/12/07). Written informed consent to participate in this study was provided by the participants’ legal guardian/next of kin.

## Author Contributions

JW, SC, and JG designed this study. JG, MC, YG, and YuL performed the experiments. JG, YaL, and JW wrote the manuscript. All the authors discussed and edited this manuscript.

## Conflict of Interest

The authors declare that the research was conducted in the absence of any commercial or financial relationships that could be construed as a potential conflict of interest.

## Footnotes

1 http://fcon_1000.projects.nitrc.org/indi/abide
